# Evaluation of macular neovascularization activity in pathological myopia: a comparison between optical coherence tomography and OCT-angiography

**DOI:** 10.3389/fmed.2023.1166271

**Published:** 2023-09-14

**Authors:** Xin Li, Ruixia Jing, Xue Li, Zhen Wang

**Affiliations:** ^1^Department of Ophthalmology, Central Hospital Affiliated to Shandong First Medical University, Jinan, China; ^2^Department of Ophthalmology, Shandong First Medical University, Jinan, China

**Keywords:** macular neovascularization, pathological myopia, optical coherence tomography, OCT-angiography, activity

## Abstract

**Purpose:**

The purpose of this study was to suggest a novel approach to assessing the activity of macular neovascularization (MNV) in pathological myopia (PM) by comparing optical coherence tomography (OCT) with OCT-angiography (OCT-A).

**Methods:**

The Zeiss Cirrus HD-5000 was used to obtain OCT and OCT-A images of PM-MNV. The objective was to examine the characteristics of PM-MNV lesions and investigate the relationship between PM-MNV activity and changes in retinal structure in 54 patients (54 eyes). To analyze the OCT parameters associated with PM-MNV activity and their clinical significance in terms of sensitivity and specificity, we used OCT-A as a reference.

**Results:**

This study included 72 patients (72 eyes), of whom 54 had good image quality and were considered for analysis. The study evaluated various OCT characteristics of MNV lesions, including the elevation of an external limiting membrane (ELM), ellipsoidal zone (EZ), retinal pigment epithelium (RPE) elevation, and EZ/RPE interruption, to identify possible parameters associated with PM-MNV activity. The interobserver consistency was found to be almost perfect. In the evaluation of PM-MNV activity, the sensitivity of ELM elevation, EZ interruption, and RPE interruption was found to be 66.7% (low), 88.4% (high), and 95.6% (high), respectively. However, the specificity was found to be 71.4% (moderate), 71.4% (moderate), and 25.4% (poor), respectively. This indicates that the current evaluation methods are not accurately assessing PM-MNV activity. We developed a new comprehensive method that used EZ interruption as the primary parameter and ELM elevation and RPE interruption as secondary parameters to evaluate PM-MNV activity with a sensitivity of 97.8% and a specificity of 85.4%.

**Conclusion:**

In PM-MNV, a novel comprehensive diagnostic method combining EZ interruption, ELM elevation, and RPE interruption might be a valuable indicator to evaluate PM-MNV activity.

## Introduction

Complications of pathological myopia (PM) are a major cause of visual impairment and blindness, especially in Asia (1). Macular neovascularization (MNV) represents the most frequent complication of PM, which frequently results in quick central vision loss (2). PM-MNV lesions has been reported to occur in 5.2%−11.3% of individuals with PM, accounting for 62% of MNV patients below the age of 50 years (3, 4). Without treatment, PM-MNV lesions has a very poor prognosis and may form scars with varying degrees of macular atrophy; within 5 years, in many cases, visual acuity would be below 20/200, which severely influences the life quality of young and middle-aged people (5, 6).

Anti-vascular endothelial growth factor (Anti-VEGF) has been the first-line option for PM-MNV (7, 8). It is essential to monitor MNV activity during anti-VEGF retreatment, with fundus fluorescein angiography (FFA) and indocyanine green angiography (ICGA) as the gold standards for diagnosis and monitoring of MNV activity (9–11). However, they are invasive and time-consuming, placing the subject at risk of side effects (12). Optical coherence tomography-angiography (OCT-A) is a non-invasive and high-speed imaging technique that has been introduced into clinical practice. It can visualize retinal and choroidal vessels, making it a useful tool for diagnosing PM-MNV (4). Li et al. (9) have demonstrated that the morphological features of lesions on OCTA can be used as a criterion to evaluate PM-MNV activity. However, they believed that patients with low vision and adverse retinal vascular perfusion might have been excluded for poor image quality due to poor fixation. This exclusion could potentially introduce bias into the study results.

Optical coherence tomography (OCT) has played a crucial role in the development of OCT-A. Over the past few decades, OCT has been instrumental in guiding the diagnosis and monitoring of polypoidal choroidal neovascularization (PM-MNV). Changes in best-corrected visual acuity (BCVA), along with the presence or absence of subretinal fluid (SRF) or intraretinal fluid (IRF), have been used as the criteria for retreatment (13, 14). However, PM-MNV has smaller lesions and fewer SRF and IRF than neovascular age-related macular degeneration (nAMD), resulting in limitations to OCT functionality (15). As OCT can visualize retinal structural changes, Ding et al. (16) have proposed that external limiting membrane (ELM) interruption and retinal pigment epithelium (RPE) elevation are valuable indicators for diagnosing and monitoring PM-MNV activity, as OCT can visualize retinal structural changes. However, the inclusion of poor image quality in their study may have affected the experimental results. Therefore, further exploration is necessary to determine indicators for monitoring PM-MNV activity by OCT.

The objective of this study was to assess the changes in the retinal structure of PM-MNV before and after anti-VEGF treatment, which included ELM, ellipsoidal zone (EZ), and RPE, with the aim of exploring the sensitivity and specificity of OCT in evaluating PM-MNV activity as well as comparing with OCT-A.

## Methods

This retrospective study was approved by the institutional review board of the Central Hospital Affiliated to Shandong First Medical University, Jinan, China and was conducted in accordance with the tenets of the Helsinki Declaration. Because this study was retrospective, the review board waived the requirement for written informed consent. Moreover, all analysis data were anonymized.

### Patients

This study included patients diagnosed with PM-MNV who received anti-VEGF therapy and were followed up for at least 6 months in the Central Hospital Affiliated to Shandong First Medical University from January 2021 to January 2023. The diagnosis of PM-MNV was confirmed by two experienced ophthalmologists (ZW and XL) for all patients included in the study.

The inclusion criteria were as follows: (1) the patients had to be above 18 years and below 55 years; (2) refractive error (RE) should be <-6.0 D and axial length (AL) should be >26.5 mm; (3) the fundus must exhibit characteristic retinal and choroidal changes, including conditions such as Fuchs' spots, macular map-like atrophy, and optic disc tilt (17).

The exclusion criteria were as follows: (1) interference of refractive media opacification with image quality; (2) presence of other MNV diseases such as nAMD, idiopathic MNV, and pachychoroid neovascular diseases; (3) history of another ocular disease, such as trauma, malignant glaucoma, and retinal choroidal disease.

In this study, only one eye from each patient was included. For patients with bilateral PM-MNV, the eye with the first treatment was prioritized, while for patients who started treatment in both eyes simultaneously, the eye with a longer follow-up was selected. Every patient performed a comprehensive ophthalmic examination at the visit, including BCVA (standard logarithmic visual acuity chart measurement and conversion to LogMAR for statistical analysis), slit lamp bio-microscopy, indirect ophthalmoscopy, OCT, and OCT-A (Carl Zeiss Meditec, Dublin, CA) examination. To standardize diagnostic criteria and improve follow-up rates, we used only OCT and OCT-A examinations, not FFA and ICGA examinations. OCT and OCTA images were acquired using equipment with a Zeiss Cirrus HD-5000 with an 840 nm center wavelength, 5 μm resolution, a scanning depth of 2.0 mm, and a scanning mode of A-scan at 68,000 scans per second. OCT images were acquired using Macular Cube 512 × 128 mode, and OCT-A images were acquired using angiography 3 × 3 mm and angiography 6 × 6 mm modes, which were performed by an ophthalmologist (RJ) skilled at operating this device. Tracking mode was selected for each scan, with good image quality according to the manufacturers' recommendations ≧7.

### Images

**Figure 1:**
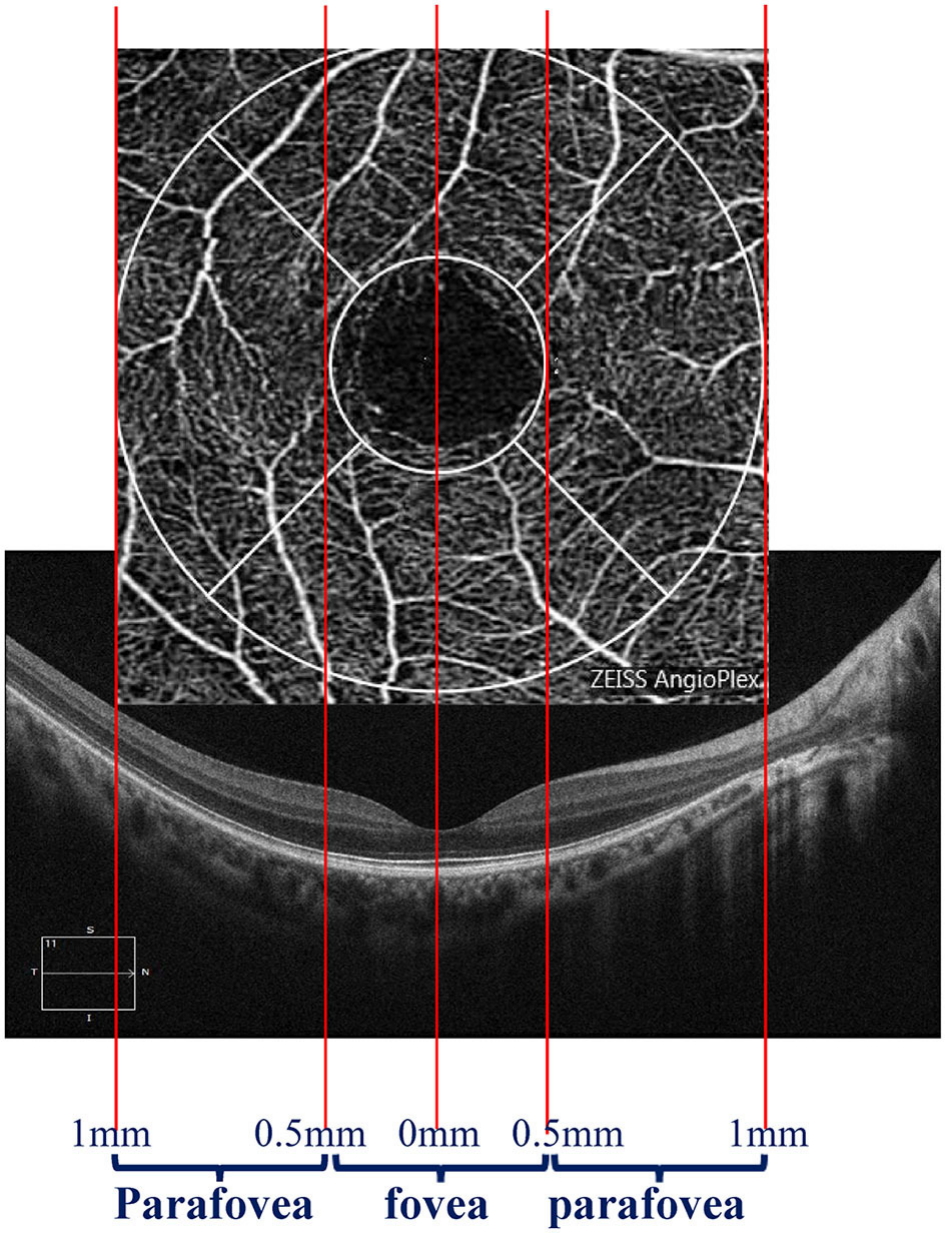
Diagram displaying the zone of interest. The fovea zone was defined by a circle with a 1-mm diameter. The parafovea zone was defined by a ring with a 0.5–1-mm radius in the center of the fovea zone.

**Figure 2:**
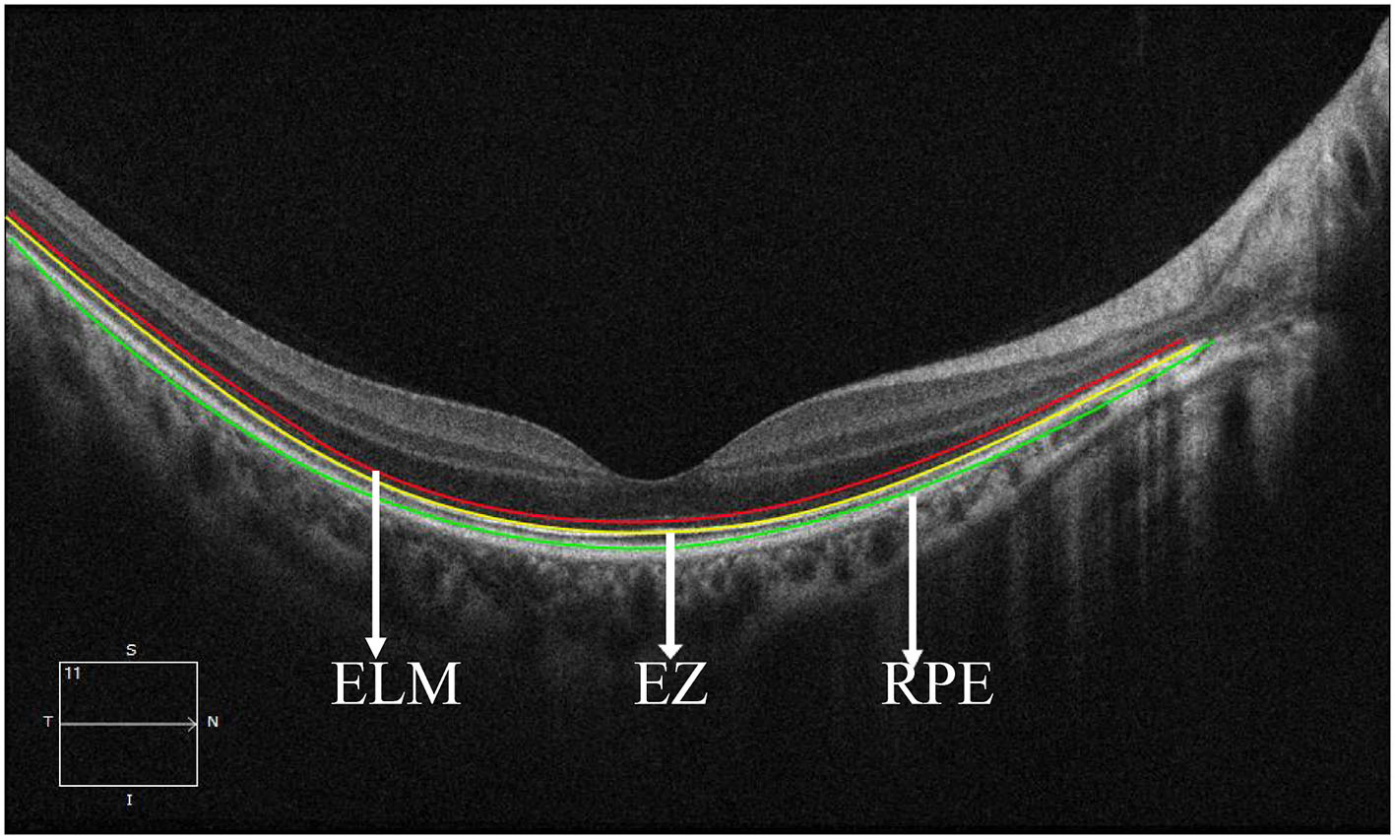
Retinal structure images on optical coherence tomography. The red line showed the external limiting membrane (ELM). The yellow line shows the ellipsoidal zone (EZ). The green line showed the retinal pigmental epithelium (RPE).

**Figure 3:**
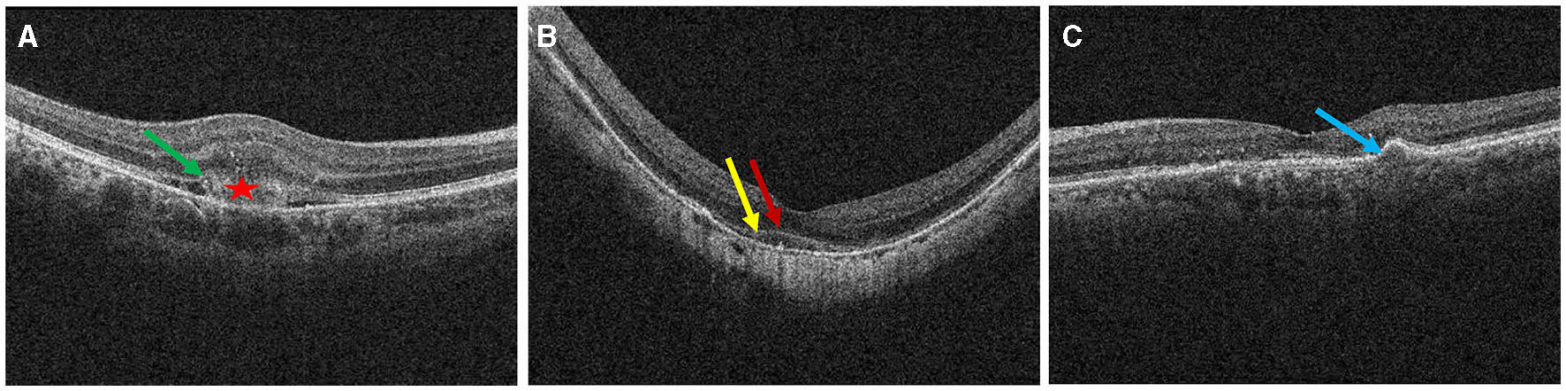
Definition of outer retinal characteristics associated with MNV activity using OCT. Subretinal hyperreflective exudation was defined as the presence of areas of high reflectivity that are located outside the RPE but within the retinal pigment epithelium [⋆ in **(A)**]. EZ/ELM interruption was defined as the absence or discontinuity of EZ/ELM hyperreflective lines above MNV lesions [**(A)**, green arrow]. EZ/ELM elevation was defined as elevated EZ/ELM hyperreflective lines above MNV lesions **(B)** (EZ, yellow arrow; ELM, red arrow). RPE elevation was defined as a highly reflective band over the MNV lesions on OCT and was continued with the RPE monolayer [**(C)**, blue arrow]. RPE interruption was identified in **(A)**.

We divided the macular zone into two subzones (Figure 1): (1) the fovea zone (1 mm in diameter from the fovea) and (2) the parafovea zone (1–3 mm in diameter from the fovea). The images were acquired centered on the fovea at each visit to observe the RPE/EZ/ELM structure on OCT (Figure 2). RPE elevation was defined as a hyperreflective line of monolayer elevation above the MNV lesion. EZ/ELM elevation was defined as elevated EZ/ELM hyperreflective lines above MNV lesions. RPE/EZ/ELM interruption was defined as the absence or discontinuity of EZ/ELM hyperreflective lines above MNV lesions (Figure 3).

**Figure 4:**
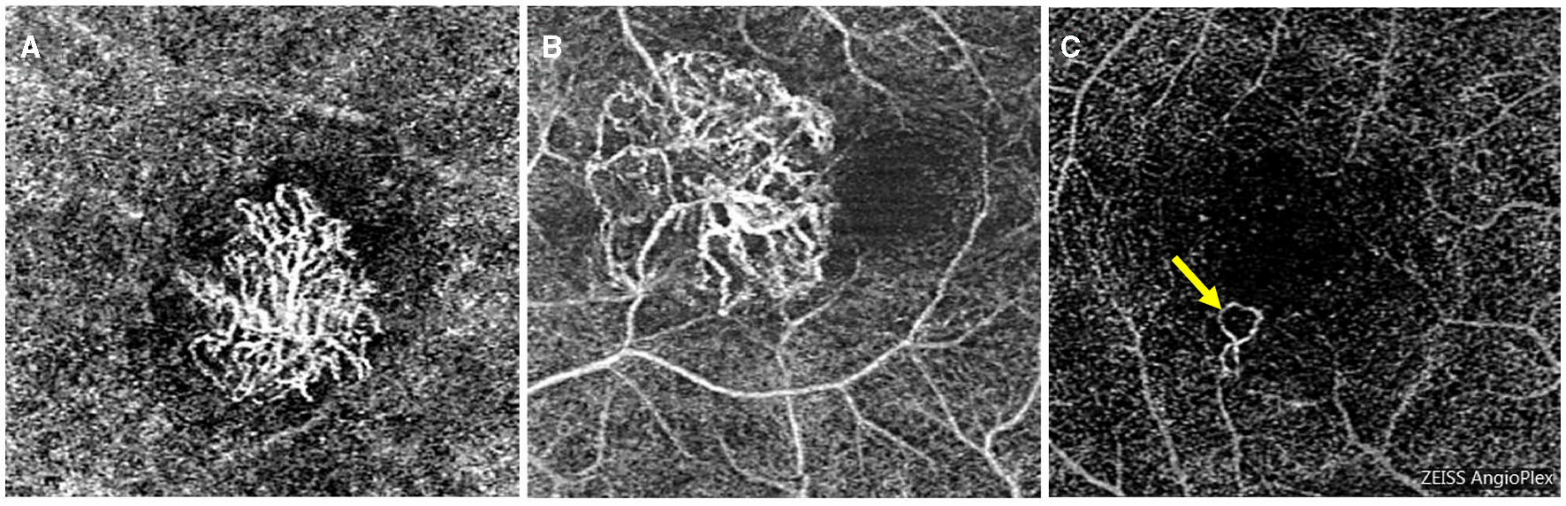
Optical coherence tomography angiography images of macular neovascularization (MNV) in pathological myopia (PM). OCT-A image of PM-MNV, which can clearly see MNV morphological characteristics, including its primary appearance as medusa or sea-fan shaped **(A, B)**, numerous microcapillaries **(B, C)**, and the presence of loops/anastomoses [**(C)**, yellow arrow].

Based on previous studies, the presence of subretinal hyperreflective exudation on OCT was an indication of an active lesion in MNV (18). Additionally, the presence of macular hemorrhage alongside neovascularization on OCT-A was also indicative of MNV activity (19, 20). PM-MNV activity on OCT-A images showed (1) a primary appearance as medusa or sea-fan shaped (Figures 4A, B); (2) numerous microcapillaries (Figures 4B, C); and (3) the presence of loops/anastomoses (Figure 4C).

Two ophthalmologists (RJ and XL) independently reviewed the images, including RPE, EZ, and RPE structure, as well as PM-MNV activity. Intraobserver and interobserver reliability were analyzed. If there was a disagreement between the two observers, we carefully examined each OCT-A image to determine the presence or absence of MNV lesions. We then compared the presence and severity of each biomarker to determine the extent of active MNV, prioritizing biomarkers with greater sensitivity and specificity. If a consensus could not be reached through discussion, a third higher-level ophthalmologist (ZW) was consulted for an independent assessment. Based on their opinion, a final decision was made.

### Adjustment for image magnification

To estimate accurate measurements, the original images were scaled using the Littman and modified Bennett formulae (21, 22). The Bennett formula calculates the magnification factor (*q*) related to the eye (21). The axial length (*x*) can be used to determine *q* using the formula: *q* = *0.01306* × *(x – 1.82)* (22).

### Statistical analysis

Statistical analysis was performed using the Statistical Package for the Social Sciences (version 26.0, IBM SPSS). The Shapiro–Wilk test was used to assess whether quantitative data obeyed a normal distribution. The variables obeying normal distribution were expressed as mean ± standard deviation. The data obeying a skewed distribution was expressed as the median (interquartile range, IQR). Categorical variables were tested with Fisher's exact test. Normal distribution data were analyzed using a one-way analysis of variance (ANOVA). Cohen's k-analysis was used to evaluate the agreement between two observers. The diagnostic sensitivity, specificity, positive predictive value, negative predictive value, agreement, and Youden index of PRE/EZ/ELM parameters were calculated by Cohen's *k*-analysis (23). The Kappa agreement meaning is as follows: <0.20 is poor, 0.21–0.40 is fair, 0.41–0.60 is moderate, 0.60–0.80 is stronger, and more than 0.80 is almost perfect. Findings with *p*-values <0.05 were considered statistically significant.

## Results

### Study population and clinical characteristics

In 72 PM-MNV eyes, OCT and OCT-A images were independently reviewed by two retinal experts, while 18 eyes were excluded due to unsatisfactory image quality. Among these 18 PM-MNV eyes, 10 were excluded due to poor image quality caused by posterior scleral staphyloma, and eight were excluded due to retinal splitting. Finally, 54 patients (54 eyes) with a mean age of 43.28 ± 10.39 (25–54) years were included, among whom 41 (75.93%) patients were female. In total, 32 right and 22 left eyes were analyzed, with a mean AL of 28.26 ± 0.99 mm, a mean RE of −14.96 ± 2.11 D, and a mean BCVA (LogMAR) of 1.02 ± 0.27. The average number of injections was 1.94 ± 0.79. Among all PM-MNV patients, seven eyes (12.97%) were located in the parafovea, and nine eyes (16.67%) were type 1 MNVs. Macular hemorrhage was present in 20 eyes. Complete demographic and clinical data are presented in Table 1. All patients received conbercept using the 1+ pro re nata (PRN) scheme. All 54 patients underwent OCT and OCT-A at baseline and in the third month. Thus, the images from 108 examinations were analyzed. According to OCT-A, 45 patients with active PM-MNV lesions and 63 patients with inactive PM-MNV lesions were found during the follow-up period. The demographic and clinical characteristics of active and inactive PM-MNV were not statistically significant (*p* > 0.05) (Table 2). Subretinal hyperreflective exudation was observed on OCT images in a total of 44 patients. Among these patients, 40 had active MNV, while 4 had inactive MNV (*p* < 0.001; Table 2). Macular hemorrhage was present in 20 eyes, of which 18 eyes were in the active group (*n* = 45, 40%) and two eyes were in the inactive group (*n* = 63, 3.17%; *p* < 0.001; Table 2).

**Table 1:** PM-MNV patients' demographic and clinical characteristics.

**No. of eyes, *n***	**54**
Age, year, median (IQR)	47 (25–54)
Sex, male/female, *n*	13/41
OD/OS, *n*	32/22
AL (mm), mean ± SD	28.26 ± 0.99
Refractive error (D), mean ± SD	−14.96 ± 2.11
BCVA (LogMAR), mean ± SD	1.02 ± 0.27
MNV type, type 1/type 2, *n*	9/45
Subfoveal MNV/parafoveal MNV, *n*	7/47
Macular hemorrhage, *n*	20
Number of injections, *n*, mean ± SD	1.94 ± 0.79

PM, pathological myopia; MNV, macular neovascularization; IQR, interquartile range; AL, axial length; SD, standard deviation; BCVA, best corrected visual acuity; LogMAR, logarithm of the minimum angle of resolution.

**Table 2:** Comparison of the demographic and clinical characteristics of active and inactive PM-MNV lesions.

**Characteristics**	**Active**	**Inactive**	***P*-value**
No. of eyes, *n*	45	63	
Age, year, mean ± SD	45.33 ± 6.89	45.33 ± 6.89	0.542^a^
Sex, male/female, *n*	13/42	13/50	0.836^b^
OD/OS, *n*	24/21	41/22	0.339^b^
AL (mm), mean ± SD	28.47 ± 1.41	28.47 ± 1.41	0.922^a^
Refractive error (D), mean ± SD	−12.58 ± 2.89	−12.58 ± 2.29	0.439^a^
BCVA (LogMAR), mean ± SD	1.02 ± 0.27	1.02 ± 0.27	0.997^a^
Subretinal hyperreflective exudation, *n* (%)	40 (88.89)	4 (6.35)	**<0.001** ^**b**^
Macular hemorrhage, *n* (%)	18 (40)	2 (3.17)	**<0.001** ^**b**^
**OCT-A features**			
Medusa/sea-fan shaped, *n* (%)	31 (68.89)	7 (11.11)	**<0.001** ^**b**^
Tiny capillaries, *n* (%)	38 (84.44)	8 (12.70)	**<0.001** ^**b**^
Loop or anastomoses, *n* (%)	34 (75.56)	5 (7.94)	**<0.001** ^**b**^
Two or more features, *n* (%)	28 (62.22)	3 (4.76)	**<0.001** ^**b**^

PM, pathological myopia; MNV, macular neovascularization; IQR, interquartile range; AL, axial length; SD, standard deviation; BCVA, best corrected visual acuity; LogMAR, logarithm of the minimum angle of resolution.

^a^ANOVA test.

^b^Fisher exact text.

P with statistical significance was shown in boldface.

### OCT-A features of active and inactive PM-MNV

The overall appearance of the MNV lesions was defined as medusa or sea fan in 38 eyes, including 31 in the active group (*n* = 45, 68.89%) and seven in the inactive group (*n* = 63, 11.11%; *p* < 0.001). Tiny capillaries were found in 46 eyes, including 38 in the active group (*n* = 45, 84.44%) and eight in the inactive group (*n* = 63, 12.70%; *p* < 0.001). Loops or anastomoses were identified in 39 eyes, with 34 in the active group (*n* = 45, 75.56%) and five in the inactive group (*n* = 63, 7.94%; *p* < 0.001). Two or more features were considered in 31 eyes, including 28 in the active group (*n* = 45, 62.22%) and three in the inactive group (*n* = 63, 4.76%; *p* < 0.001; Table 2).

### Interobserver agreement regarding retinal structural characteristics on OCT

All OCT images were analyzed independently two times by two observers. The kappa values for subretinal hyperreflective exudation, SRF, IRF, ELM elevation, EZ elevation, EZ interruption, RPE elevation, and RPE interruption between two observers were 0.906, 0.509, 0.254, 0.814, 0.832, 0.83, 0.761, and 0.721, respectively. IRF showed fair agreement, SRF showed moderate agreement, and ELM elevation, EZ elevation, subretinal hyperreflective exudation, and EZ interruption showed almost perfect agreement. RPE elevation and RPE interruption showed stronger agreement (Table 3).

**Table 3:** Interobserver agreement regarding retinal structural characteristics on OCT.

**Characteristic**	**Observer 1**	**Observer 2**		**Agreement (%)**	**κ value**	**Strength of agreement**
		+	**–**			
Subretinal hyperreflective exudation	+	45	3	95.37	0.906	Very good
	–	2	58			
SRF	+	12	8	85.2	0.509	Moderate
	-	8	80			
IRF	+	12	10	71.3	0.254	Fair
	–	21	65			
ELM elevation	+	46	6	90.7	0.814	Very good
	–	4	52			
EZ elevation	+	45	5	91.7	0.832	Very good
	–	4	54			
EZ interruption	+	42	4	91.7	0.83	Very good
	–	5	57			
RPE elevation	+	14	4	93.5	0.761	Good
	–	3	87			
RPE interruption	+	87	5	92.6	0.721	Good
	–	3	13			

SRF, subretinal fluid; IRF, intraretinal fluid; OCT, optical coherence tomography; ELM, external limiting membrane; EZ, ellipsoidal zone; RPE, retinal pigmental epithelium.

### Inter-observer agreement regarding the OCT-A features

Three descriptive features of OCT-A, “A (appearance)—T (Tiny capillary)—A (anastomoses/loops),” were used in this study: overall appearance of the MNV lesion, tiny capillaries, and the presence of anastomoses/loops. The interobserver agreement of A-T-A was substantial to almost perfect, with kappa values of 0.863, 0.906, and 0.881, respectively. The interobserver agreement of two or more features was substantial to almost perfect, with kappa values of 0.822 (Table 4).

**Table 4:** Interobserver agreement regarding the OCT-A features.

**Features**	**Observer 1**	**Observer 2**		**Agreement (%)**	**κ value**	**Strength of agreement**
		+	**–**			
Medusa/sea-fan shaped	+	38	4	93.52	0.863	Very good
	–	3	63			
Tiny capillaries	+	45	2	95.37	0.906	Very good
	–	3	58			
Loop or anastomoses	+	37	4	94.4	0.881	Very good
	–	2	65			
Two or more features	+	28	3	92.60	0.822	Very good
	–	5	72			

OCT-A, optical coherence tomography angiography.

### Specificity and sensitivity of SRF/ELM/EZ/RPE compared with OCT-A for the diagnosis of PM-MNV activity

The relationship between IRF and MNV activity was not analyzed due to the fair agreement between IRF and the two observers. In 14 of 108 PM-MNV patients, SRF present on OCT was consistent with activity on OCT-A, with a kappa value of 0.308, a sensitivity of 31.1% (poor), a specificity of 96.8% (high), and the agreement being fair (*p* = 0.079; Table 5). Evaluation of PM-MNV activity included the assessment of ELM/EZ/RPE elevation and EZ/RPE interruption. In 75 of 108 PM-MNV patients, ELM structural status on OCT was consistent with the activity on OCT-A, with a kappa value of 0.377, sensitivity of 66.7% (fair), specificity of 71.4% (moderate), and the agreement being fair (*p* < 0.001; Table 5), indicating that ELM elevation may be a biomarker in PM-MNV activity. However, there was no agreement among the 33 patients with ELM elevation but PM-MNV inactivity in 18 of 33 patients (Table 5).

**Table 5:** Retinal structural characteristics on OCT relationship with PM-MNV activity.

	**Active MNV on OCT-A (*n* = 45)**	**Inactive MNV on OCT-A (*n* = 63)**	**SE (%)**	**SP (%)**	**PPV (%)**	**NPV (%)**	**κ value**	***P*-value**	**Youden index**
**SRF**									
+	14	2	31.1	96.8	87.5	66.3	0.308	0.079	0.279
–	31	61							
**ELM elevation**									
+	30	18	66.7	71.4	62.5	75.0	0.377	**<0.001**	0.381
–	15	45							
**EZ elevation**									
+	25	23	55.6	63.5	44.4	66.7	0.189	0.05	0.191
–	20	40							
**EZ interruption**									
+	38	19	84.4	71.4	67.9	86.5	0.54	**<0.001**	0.558
–	7	44							
**RPE elevation**									
+	8	9	17.8	85.7	47.1	59.3	0.038	0.623	0.035
–	37	54							
**RPE interruption**									
+	42	46	95.6	25.4	47.8	88.9	0.183	**0.004**	0.367
–	3	17							
**A primary criterion and two secondary criteria**									
+	44	8	97.8	87.3	84.6	98.2	0.832	**<0.001**	0.851
–	1	55							

SRF, subretinal fluid; OCT, optical coherence tomography; PM, pathological myopia; MNV, macular neovascularization; OCT-A, OCT-angiography; SE, sensitivity; SP, specificity; PPV, positive predictive value; NPV, negative predictive value; ELM, external limiting membrane; EZ, ellipsoidal zone; RPE, retinal pigmental epithelium.

P with statistical significance was shown in boldface.

A total of 48 of 108 PM-MNV patients were observed to have EZ elevation on OCT, 25 of 48 patients showed active on OCTA, and 23 of 48 patients showed inactive on OCT-A. The kappa value was 0.189, sensitivity was 55.6% (fair), specificity was 63.5% (fair), and the agreement was poor (*p* > 0.05; Table 5), indicating that EZ elevation was not a biomarker of PM-MNV activity. However, 57 of 108 PM-MNV patients observed EZ interruption on OCT, 38 of 57 patients showed active OCT-A, and 19 of 57 patients showed inactive OCT-A, with the kappa value of 0.54, the sensitivity of 84.4% (high), and the specificity of 71.4% (moderate). There was moderate agreement (*p* < 0.001; Table 5), indicating that EZ interruption might be a biomarker of PM-MNV activity.

Moreover, 17 of 108 patients with PM-MNV were observed with RPE elevation on OCT, 8/17 showed active on OCT-A, and 9/17 showed inactive on OCT-A. The kappa value was 0.038; sensitivity was 17.8% (low), specificity was 85.7% (high), and agreement was poor (*p* > 0.05), indicating that RPE elevation was not a biomarker of PM-MNV activity. However, 88/108 PM-MNV patients with RPE interruption were observed on OCT, 42/88 showed active on OCTA, and 46/88 showed inactive on OCT-A, with a kappa value of 0.183, a sensitivity of 95.6% (high), a specificity of 25.4% (poor), and the agreement being poor (*p* < 0.001; Table 5), suggesting that RPE interruption might also be a rational biomarker of PM-MNV activity.

### New strategies for OCT evaluating PM-MNV activity

Using ELM elevation to evaluate PM-MNV activity had fair sensitivity (66.7%), moderate specificity (71.4%), and fair agreement (kappa = 0.377). Using RPE interruption to evaluate PM-MNV activity had high sensitivity (95.6%), poor specificity (25.4%), and poor agreement (kappa = 0.183). On the contrary, using EZ interruption to evaluate PM-MNV activity had high sensitivity (88.4%), moderate specificity (71.4%), and moderate agreement (kappa = 0.54). Therefore, we developed a new comprehensive program consisting of a primary parameter (EZ interruption) and two secondary parameters (ELM elevation and RPE interruption). At first, the primary parameter (EZ interruption) was evaluated. If “yes” (*n* = 57), the lesion was probably active (*n*= 38). If “no” (*n* = 51), two secondary parameters were evaluated. If the lesion had RPE interruption or ELM elevation (6/51), it was probably active. Using this new method to evaluate PM-MNV activity, ultimate sensitivity and specificity reached 97.8 and 87.4%, respectively. The ultimate agreement was as high as 91.7%, with a kappa value of 0.832, which was considered an almost perfect agreement (Table 5).

## Discussion

This study assessed the structural characteristics of PM-MNV using OCT and analyzed the relationship between changes in these parameters and PM-MNV activity. Additionally, the study compared PM-MNV activity to OCT-A. The result suggested that ELM elevation and EZ/RPE interruption serve as indicators to evaluate PM-MNV activity. Furthermore, we have designed a novel, comprehensive method for evaluating PM-MNV activity. In detail, one primary parameter (EZ interruption) and two secondary parameters (ELM elevation and RPE interruption) were used.

While our study introduces a novel diagnostic method for assessing PM-MNV activity, it is important to note that we did not incorporate FFA in our assessment of MNV activity. This limitation prevented us from observing the real-time evolution of MNV lesions and assessing vascular leakage, which was also one of the gold standards for evaluating MNV according to previous research (24). The absence of this observation may result in an inaccurate assessment of MNV activity. However, FFA is an invasive and time-consuming procedure that may lead to systemic adverse effects to varying degrees (9, 10). In our study, we addressed this limitation by employing OCT and OCT-A, which effectively compensated for the lack of FFA and provided valuable insights.

Optical coherence tomography (OCT) has proven to be effective in identifying retinal structural changes, detecting SRF/IRF, and conducting both quantitative and qualitative analyses of the retina and choroid, improving the diagnosis of the disease (25, 26). Nevertheless, despite its effectiveness, PM-MNV behaves similarly to classical MNV (type 2) when assessed using OCT. However, using SRF/IRF to guide anti-VEGF treatment of PM-MNV is difficult. This is because PM-MNV lesions are smaller and have fewer SRF and IRF than nAMD, and in some cases, it is difficult to determine whether IRF is present (7, 16). Parodi et al. also showed that IRF was detected in only 3.3% (1/30) of active PM-MNV at baseline and in only 15.6% (5/32) of active MNV during follow-up (27). Therefore, IRF cannot be used as a biological indicator of PM-MNV activity. Moreover, Ding et al. found that SRF has only 23.9% (poor) sensitivity for diagnosing PM-MNV activity (16). This was consistent with our study results. This suggested that retreatment criteria based on sustained SRF/IRF would seem inappropriate and insensitive for PM-MNV. Therefore, a mechanism for monitoring PM-MNV activity using OCT was unknown.

To the best of our knowledge, previous studies have found the combination of ELM interruption and RPE elevation to have high sensitivity (92.5%) and specificity (95.1%) for monitoring PM-MNV activity (16). However, our study found that RPE elevation was not an indicator of PM-MNV activity, which was contrary to the results of Ding et al. (16). The reason for this discrepancy may be that, in this study, we assessed the activity of PM-MNV solely based on the morphological features observed on OCT-A, without comparing it with FFA, which was considered the gold standard. This approach may introduce bias in the assessment of MNV activity, leading to inconsistent results compared to previous studies. In contrast, they assessed MNV activity using FFA. This may also be due to RPE elevation being frequently detected in high myopia, which was previously reported by Marchese et al. (28). Meanwhile, they also suggested that RPE humps were not correlated with any pathologic accumulation and thus should be distinguished from pathologic pigment epithelial detachment and MNV to prevent unnecessary treatment. Therefore, whether RPE elevation can be used as an indicator of PM-MNV activity needs to be confirmed by prospective studies with large samples in the future.

Previous studies have found that EZ thickness thinning is significantly associated with PM visual deterioration (29). The extent of EZ damage was also an important factor in visual acuity recovery after nAMD anti-VEGF (30). Therefore, in the current study, we analyzed the value of EZ elevation/interruption in evaluating PM-MNV activity. The results suggested that EZ elevation was not a biomarker of PM-MNV activity. However, the EZ interruption may be a biomarker of PM-MNV activity with a sensitivity of 84.4% (high) and a specificity of 71.4% (moderate). EZ elevation was not a biological indicator of PM-MNV activity, which may be due to EZ thinning in PM (29), resulting in a majority of EZ interruption in PM-MNV.

In our study, it was determined that using solely ELM elevation, EZ interruption, or RPE interruption to evaluate PM-MNV activity did not yield high sensitivity and specificity. In light of this limitation, we developed a novel comprehensive procedure that considers EZ interruption as the primary indicator and the other two as secondary indicators to identify PM-MNV activity. According to this criterion, a PM-MNV lesion with either one major sign or two minor signs was considered active. The final sensitivity and specificity of this method were found to be 97.8 and 87.4%, respectively. Therefore, we conclude that this novel procedure is easy to conduct, non-invasive, fast, and informative for screening active PM-MNV lesions and diagnosing and monitoring PM-MNV. However, the specificity was not very high compared to previous studies (9, 17). This discrepancy may be due to our not using FFA as the gold standard to evaluate PM-MNV activity, while they all used FFA.

This study had some limitations. First, PM-MNV activity was evaluated without comparison to FFA as the gold standard, which might produce either false negatives or false positives. Second, due to the limitations of OCT-A technology, OCT-A showed blood flow signals by detecting red blood cell flow rate. When the red blood cell flow rate was too slow or too fast, OCT-A could not detect the blood flow signal (neovascularization), and the image showed no perfusion or no vascular zone (31), which may lead to low detection rates and incorrect evaluation of PM-MNV activity. Third, changes to ELM/EZ/RPE structures in this study were all subjective and qualitative, which might have an impact on the experimental results. An objective and quantitative assessment will be needed in the future. Fourth, the study did not exclude type 1 MNV, which may also lead to bias in the experimental results. Finally, this study was a retrospective study with a small sample. A prospective study with a large sample size is recommended to further evaluate the effectiveness of the structural OCT diagnostic algorithm presented in predicting the activity of PM-MNV on FFA.

In conclusion, the results of our study provided a simple, fast, and accurate alternative to evaluating PM-MNV activity based on non-invasive OCT. Active PM-MNV mainly showed ELM elevation/EZ interruption/RPE interruption on OCT. A novel comprehensive procedure that included one primary parameter or two secondary parameters showed good sensitivity and specificity in evaluating PM-MNV activity based on non-invasive OCT.

## Data availability statement

The raw data supporting the conclusions of this article will be made available by the authors, without undue reservation.

## Ethics statement

The studies involving humans were approved by the Institutional Review Board of the Central Hospital Affiliated to Shandong First Medical University. The studies were conducted in accordance with the local legislation and institutional requirements. The Ethics Committee/Institutional Review Board waived the requirement of written informed consent for participation from the participants or the participants' legal guardians/next of kin because this study was retrospective. Written informed consent was not obtained from the individual(s) for the publication of any potentially identifiable images or data included in this article because this study was retrospective. Moreover, all analysis data were anonymized.

## Author contributions

The study was designed by XiL and ZW. The data was collected and analyzed by RJ, XiL, and XuL. The manuscript was written by XiL, RJ, and ZW. All authors have discussed the findings and commented on the manuscript. All authors contributed to the article and approved the submitted version.
